# The Pivotal Role of NF-kB in the Pathogenesis and Therapeutics of Alzheimer’s Disease

**DOI:** 10.3390/ijms23168972

**Published:** 2022-08-11

**Authors:** Emily Sun, Aishat Motolani, Leonardo Campos, Tao Lu

**Affiliations:** 1 Department of Pharmacology & Toxicology, Indiana University School of Medicine, Indianapolis, IN 46202, USA; 2 Department of Biological Sciences, Columbia University, 70 Morning Drive, New York, NY 10027, USA; 3 Department of Biochemistry & Molecular Biology, Indiana University School of Medicine, Indianapolis, IN 46202, USA; 4 Department of Medical & Molecular Genetics, Indiana University School of Medicine, Indianapolis, IN 46202, USA

**Keywords:** Alzheimer’s, drug discovery, inflammation, neurodegeneration, NF-κB

## Abstract

Alzheimer’s Disease (AD) is the most common neurodegenerative disease worldwide, with a high prevalence that is expected to double every 20 years. Besides the formation of Aβ plaques and neurofibrillary tangles, neuroinflammation is one the major phenotypes that worsens AD progression. Indeed, the nuclear factor-κB (NF-κB) is a well-established inflammatory transcription factor that fuels neurodegeneration. Thus, in this review, we provide an overview of the NF-κB role in the pathogenesis of AD, including its interaction with various molecular factors in AD mice models, neurons, and glial cells. Some of these cell types and molecules include reactive microglia and astrocytes, β-secretase, APOE, glutamate, miRNA, and tau protein, among others. Due to the multifactorial nature of AD development and the failure of many drugs designed to dampen AD progression, the pursuit of novel targets for AD therapeutics, including the NF-κB signaling pathway, is rising. Herein, we provide a synopsis of the drug development landscape for AD treatment, offering the perspective that NF-κB inhibitors may generate widespread interest in AD research in the future. Ultimately, the additional investigation of compounds and small molecules that target NF-κB signaling and the complete understanding of NF-κB mechanistic activation in different cell types will broaden and provide more therapeutic options for AD patients.

## 1. Introduction

Neurodegenerative diseases are a group of disorders involving the progressive deterioration and loss of nerve cells. Currently, Alzheimer’s Disease (AD) is the most common neurodegenerative disease worldwide. The National Health Service (NHS) reports that AD affects around one in six individuals over the age of 80 [1,2]. Considering the aging population, the overall prevalence of AD is projected to sharply increase in coming years.

AD is a form of dementia in which there is marked impaired cognitive function, most identifiably and commonly associated with deteriorating memory. Considering the influence of dementias on memory, one of the major hallmarks of AD is neurodegeneration in the hippocampus. As the disease progresses however, reductions in cortical pyramidal neurons are observed in widespread portions of the temporal, parietal, and, eventually, frontal cortices. With this, impairments range from higher level cognitive deficits to changes in emotional state and personality. Although the etiology of AD is not definitively known, the prevailing theory describes the onset of AD as the result of the accumulation of β-amyloid plaques in which amyloid proteins aggregate and nucleate into a β-sheet. This leads to the induction of an inflammatory response by microglia in the corresponding area to remove such plaques, resulting in nearby neurodegeneration [2]. Although AD may significantly compromise quality of life for patients and loved ones, there is currently no cure for this disorder. Furthermore, AD is caused by a combination of genetic, lifestyle, and environmental contributors: this multifactorial nature means that the exact etiologies for AD remain unknown. Thus, there is an urgent need to elucidate the underlying pathways which contribute to the development and progression of AD.

## 2. The NF-κB Family and Its General Role in Inflammation

The nuclear factor-κB (NF-κB) is composed of a family of five transcription factors involved in various cellular processes, and it is particularly notorious for its role in mediating inflammatory responses. This family consists of NF-κB1 (p105/p50), NF-κB2 (p100/p52), RelA (p65), RelB, and c-Rel. NF-κB activation promotes the transcription of target genes, many of which are proinflammatory. The NF-κB signaling is activated via two distinct pathways: the canonical and noncanonical pathways. Notably, the canonical pathway is highly studied and plays a critical role in inflammatory responses, a key characteristic in AD development. In an inactive state, the p65/p50 dimers in the canonical NF-κB pathway are sequestered in the cytoplasm by IκBα. Upon exposure to proinflammatory stimuli such as cytokines, pathogens, and danger-associated molecular patterns, the p65/p50 dimers are released from IκBα due to the phosphorylation cascade that results in the proteasomal degradation of IκBα. Subsequently, p65/p50 translocates into the nucleus, where it binds to its cognate κB motif, leading to the activation and expression of NF-κB target genes [3]. On the other hand, the non-canonical pathway is activated through a subset of Tumor Necrosis Factor Receptor (TNFR) superfamily members, leading to the activation of NF-κB inducing kinase (NIK). NIK phosphorylates IκB kinase alpha (IKKα), which phosphorylates the C-terminal of p100 to generate p52. Following the phosphorylation cascade, p52/RelB translocates into the nucleus, triggering the expression of NF-κB target genes that play a role in immune cells’ development [4]. 

As a result of NF-κB’s extensive involvement in cellular inflammatory responses, it has become an attractive target for research on inflammatory diseases. In the AD brain, Toll-like Receptors (TLRs) are overexpressed on microglia and neurons. TLRs mainly activate the canonical NF-κB signaling pathway, leading to the expression of proinflammatory factors [5]. Microglial activation is one of the early events that lead to AD development, given that the primary function of microglia in the brain is the protection from pathogens and the clearance of cellular debris, including amyloid beta (Aβ) plaque formation. Thus, the activation of NF-κB signaling and consequent release of cytokines and chemokines from microglia results in chronic inflammation observed in AD [6]. As such, the role of NF-κB in AD is an important topic that warrants more attention in the field of AD research. Herein, we discuss the contribution of NF-κB signaling to AD pathology and provide an overview of current drugs approved/in development for AD, including NF-κB inhibitors.

## 3. Role of NF-κB in AD

As aforementioned, neuroinflammation, oxidative stress, and apoptosis are some of the key processes that contribute to the fatality of AD patients. The major histological features essential for AD diagnosis, such as formation of amyloid-beta (Aβ) plaques and neurofibrillary tangles (NFT) in neurons, can be exacerbated by inflammation perpetuated by glial cells [7]. Consequently, Aβ and NFT promotes neuronal loss and instability [8]. NF-κB is central to this vicious cycle of neurodegeneration observed in AD [9]. However, depending on the cell type and/or combination of NF-κB subunits, the activation of NF-κB can play a dual role in either neuroprotection or neurodegeneration [10]. This is evident from several studies that have shown the expression of proapoptotic genes which cause neuronal death via the transactivation of p65/p50 dimers. On the contrary, c-Rel-containing dimers mediate anti-apoptotic gene expression, thereby, promoting neuronal survival. c-Rel or p65/p50 heterodimers can be selectively activated depending on the nature of stimuli received such as IL-1β, Nerve Growth Factor (NGF), Aβ peptide, or glutamate [11,12]. 

Mechanistically, NF-κB plays a crucial role in AD pathogenesis by regulating different molecules responsible for promoting the morbidities associated with AD. Below, we will provide a synopsis of various representative factors that are involved in the activated NF-κB signaling in AD pathogenesis. 

### 3.1. NF-κB and β-Secretase in AD

**Figure 1:**
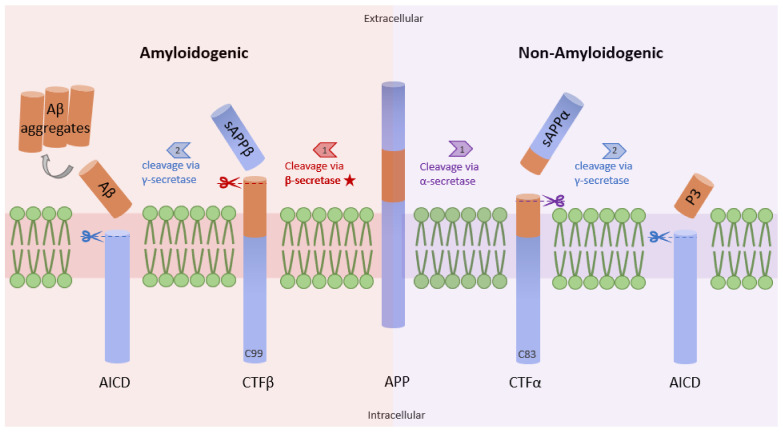
Amyloidogenic and non-amyloidogenic pathways. APP (center) can undergo proteolytic processing through two unique pathways, amyloidogenic processing (left) and non-amyloidogenic processing (right). In amyloidogenic processing, β-secretase cleaves APP, forming C99 and sAPPβ. C99 is further cleaved by γ-secretase to form amyloid beta peptides (Aβ). Importantly, the Aβ formation rate is dependent on the cleavage rate of APP by β-secretase. In non-amyloidogenic processing, APP is cleaved by α-secretase to form C83 and sAPPα, which can be further cleaved by γ-secretase, yielding p3 (adapted from [14]).

Studies have shown increased NF-κB levels in the cerebral cortex of AD patients, and this correlates with high levels of β-site amyloid precursor protein (APP) cleaving enzyme-1 (BACE1). The data from a previous study demonstrate that the p65 subunit of NF-κB binds to the κB elements on the promoter of BACE1, inducing the expression of β-secretase [13]. High levels of β-secretase facilitate the amyloidogenic pathway of APP processing, resulting in the formation of amyloid fibrils, which consequently aggregate into amyloid plaques (Figure 1) [14]. Similarly, Aβ oligomers can in turn stimulate NF-κB activation in neurons and glial cells [15]. Aβ40 peptide was shown to strongly activate the p65/p50 dimers of NF-κB and induced the expression of pro-apoptotic genes in primary and N2TN neurons. Some of the genes expressed following Aβ40 induction include Bax, p63, DcR2, and TANK (TRAF family member-associated NF-κB activator), and they all possess κB regulatory elements in their promoter region. Also, Aβ40 increased the accumulation of Aβ42 aggregates, further promoting the neuropathology cascade of AD [16]. Similarly, Aβ (25–35) peptide was shown to cause toxicity in primary neurons and cell lines through increased production of peroxides, a source of oxidative stress. This phenomenon is also accompanied by high levels of NF-κB signaling [17]. Because reactive oxygen species (ROS) are known to activate NF-κB subunits in some cases, this study suggests an indirect link between Aβ peptide-mediated toxicity and NF-κB activation. 

### 3.2. NF-κB in Reactive Microglia and Astrocytes

Considering the role of glial cells in inflammation, NF-κB signaling in reactive microglia and astrocytes has been reported to contribute to AD pathology. Bacteroides fragilis Lipopolysaccharide (BF-LPS) activated the NF-κB pathway via distinct Toll-like receptors (TLR2 and TLR4) and CD14 receptors present on microglial cell surfaces in a neuron-glial co-culture experiment. BF-LPS, among other several activators of NF-κB such as Aβ42, TNF-α, and IL-1β, exhibited the highest potency in p65/p50 dimer activation [18]. Another study demonstrated the high levels and co-localization of LPS with Aβ42. LPS was shown to promote the NF-κB-dependent transcription of cytokines, causing increased accumulation of Aβ plaques and increased tau protein hyperphosphorylation. The generation of high levels of proinflammatory cytokines, such as IL-1, IL-6, and TNF-α, damages the oligodendrocytes, promoting myelin injury [19]. This leaves the neurons vulnerable to Aβ neurotoxicity and promotes the autocrine loop required for AD progression [19]. Astrocytes, a macroglial cell, which is pivotal to maintaining brain homeostasis, also promote neurodegeneration via NF-κB, leading to Aβ42 accumulation, pro-inflammatory cytokine production, and the generation of inducible Nitric Oxide Synthase (iNOS) [20,21,22].

### 3.3. NF-κB and ApoE in AD

Additionally, apolipoprotein E allelic variants are associated with AD development, with APOEε2 causing reduced risk and APOEε4 causing increased risk in comparison to the common allele, APOEε3. Particularly, APOEε4 was discovered to inhibit Aβ clearance from the brain interstitial fluid through various mechanisms [23]. In another study, it was demonstrated that Aβ40 could activate NF-κB and lead to the increase of APOE promoter function. The regulatory region of the APOE gene has been further characterized and was shown to contain two functional κB motifs. In this study, the NF-κB inhibitor sodium salicylate was further applied to evaluate the effect of NF-κB inhibition on the promoter activity of APOE in AD. Data suggested that NF-κB-dependent APOE promoter activity was significantly decreased upon the treatment with NF-κB inhibitor [24]. Thus, considering that APOE enhances Aβ fibril formation in AD pathogenesis, the use of an NF-κB inhibitor may lessen NF-κB-induced APOE activity in the AD brain. Similarly, gene clustering analysis by Ophir et al. revealed the greater activation of NF-κB and upregulation of NF-κB-inducible genes in APOE4 mice when compared to APOE3 mice following treatment with LPS. These upregulated genes include chemokines and inflammatory cytokines like IL-1β, IL-6, CCL-3, and TNF-α, among others [25].

### 3.4. NF-κB and Glutamate in AD 

Furthermore, NF-κB is involved in Aβ oligomer-induced glutamate excitotoxicity which contributes to the AD neurodegeneration cascade. Aβ peptides have been shown to increase glutamate receptor activation with concomitant increases in intracellular calcium levels in human cerebral cortical neurons. Sustained increases in the levels of intracellular calcium is known to cause microtubule instability, increased tau phosphorylation via calcium dependent kinases, mitochondrial oxidation impairment, and ultimately increased ROS generation [26,27]. Notably, Lim and colleagues confirmed high levels of calcium and metabotropic glutamate receptor 5 (mGluR5) near Aβ plaques in the hippocampal astrocytes of AD patients. This study reveals that Aβ42 increases cytosolic calcium levels by activating calcineurin (CaN), which in turn enables the NF-κB-dependent transcription of mGluR5. It was shown that the activation of NF-κB by CaN might have occurred via the CaN dephosphorylation of B-cell lymphoma 10 (Bcl10) [28]. Bcl10 is known to activate the NF-κB pathway via ubiquitination of IKK-γ [29]. Similarly, mGluR5 staining co-localizes with the accumulated nuclear p65 subunit of NF-κB in hippocampal astrocytes, further reinforcing the link between NF-κB and glutamate in promoting AD-like pathology [28]. 

### 3.5. NF-κB and miRNAs in AD

Beyond the aforementioned factors, NF-κB also exerts its neurotoxic effect in AD via the regulation of microRNA. MicroRNAs such as miRNA-125b, miRNA-9, miRNA-155, miRNA-34a, miRNA-146a have been shown to be regulated by NF-κB [30]. Notably, miRNA-125b is the most abundant in the human brain and is highly upregulated in AD tissues [31]. NF-κB-activated mir-125b was reported to inhibit complement factor H (CFH) in human neuronal-glia cells. CFH is known to play an important role in suppressing the innate immune system by inhibiting the conversion of C3 to C3b in the complement pathway [32]. Similarly, NF-κB-induced mir-125b has been shown to silence 15-lipoxygense (15-LOX) expression, an enzyme that is important for the conversion of docohexaneoic acid to neuroprotectin D1 (NPD1) and vitamin D3 receptor (VDR). Both NPD1 and VDR are essential for protecting the brain from the toxic effects of ROS and reactive nitrogen species (RNS) [31]. Another study showed that miRNA-34a downregulates Triggering Receptor Expressed in Myeloid Cells 2 (TREM2) in the hippocampal CA1 region of AD patients. TREM2 plays a crucial role in the microglial clearance of Aβ [33]. Taken together, the aforementioned evidence suggests the expansive role of NF-κB in AD progression through the regulation of microRNA expression.

### 3.6. NF-κB and Tau Pathology in AD

Additionally, NF-κB signaling contributes to tau pathology. A study demonstrated that NF-κB can induce the expression of SET gene isoform 1, which is upregulated in AD patients’ brains [34]. SET was shown to contain a functional κB sequence in its promoter region. In the cytoplasm, SET causes the inhibition of protein phosphatase 2A (PP2A), a major tau phosphatase that prevents tau hyperphosphorylation. SET is also known to bind to the pro-apoptotic domain of APP, leading to neuronal apoptosis [34]. Comparably, a previous study observed the glycosylation of paired helical filament tau by advanced glycation end products (AGE). AGE-tau was shown to generate high levels of ROS, resulting in nuclear translocation of p65/p50 dimers and consequently increased IL-6, APP, and Aβ production in primary cortical neurons and neuroblastoma cells [35]. 

**Figure 2:**
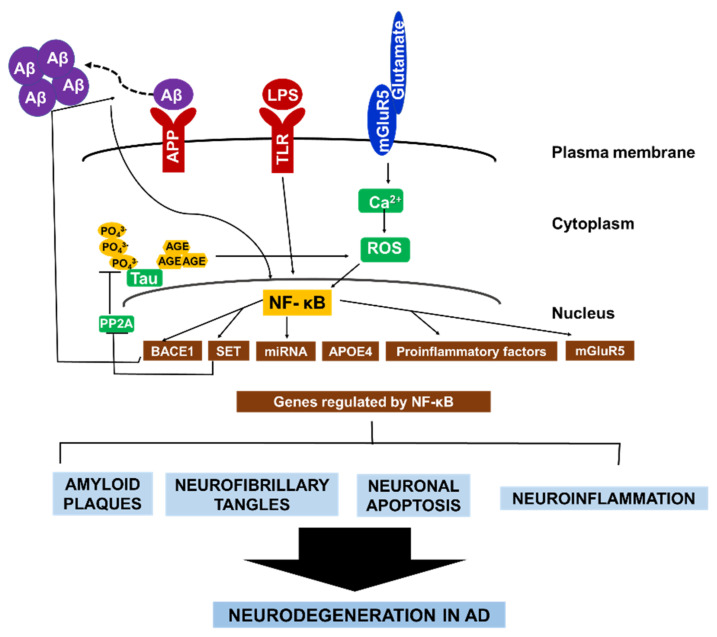
The central role of NF-κB in AD pathology. NF-κB facilitates the autocrine production of several factors that constitute AD pathology. NF-κB activates BACE1, which promotes Aβ fibril formation. Consequently, Aβ fibrils directly activate NF-κB, leading to the expression of APOE4, and mGluR5. In microglia and astrocytes. Aβ42 activates NF-κB, which induces the expression of proinflammatory factors that causes myelin injury. Additionally, NF-κB activates microRNA production which suppresses the expression of various neuroprotective factors. Similarly, the formation of hyperphosphorylated tau in AD brain is enhanced by NF-κB-dependent activation of SET, which inhibit tau’s dephosphorylation. Conversely, glycated tau triggers ROS production, leading to NF-κB activation. Collectively, the different pathways that contribute to neurodegeneration in AD are highly interconnected via continuous NF-κB activity in both neurons and glial cells.

To sum up, these aforementioned examples of NF-κB signaling and its regulation of various gene expressions in neuronal and glial cells underscores the role of NF-κB in perpetuating a cycle of neurodegeneration in AD (Figure 2). 

## 4. Overview of Drugs That Interfere with NF-κB Signaling and Other AD Treatments

Despite a century of research on AD, the exact cause of AD remains elusive, given the multitude of factors that leads to its development. Currently, the only FDA-approved treatments available to AD patients are used to manage their symptoms. These include the class of acetylcholinesterase inhibitors and N-methyl-D-aspartate receptor (NMDAR) antagonists. This is perhaps unsurprising, given that acetylcholine and NMDAR play a vital role in cognitive function and excitotoxicity, respectively [36]. Importantly, the approved NMDAR antagonist for AD, Memantine, has been shown to block the activity of NF-κB and NF-κB-dependent adhesion molecules in human brain microvascular endothelial cells. This results in the reduced migration of monocytes and decreased blood-brain barrier permeability, both of which contribute to neuroinflammation and neurodegeneration in AD [37]. 

In addition, the role of NF-κB in regulating inflammation and other processes fundamental to AD progression has made it a prime target for Non-Steroidal Anti-inflammatory Drugs (NSAIDs). For example, NSAIDs such as ibuprofen, indomethacin, and aspirin were shown to inhibit NF-κB induced expression of BACE1 in transfected HEK 293T cells [13]. Likewise, the activity of Lipoxin A4, an endogenous lipid mediator with potent anti-inflammatory properties, was triggered by aspirin, and this led to decreased AD-like pathology in mice via reduced NF-κB signaling, amongst other pathways [38]. However, a randomized trial on the effect of aspirin on enhancing impaired cognition yielded negative results [39]. Additionally, minocycline, a tetracycline derivative, downregulated Aβ levels through inhibition of the NF-κB pathway. This caused decrease in inflammatory markers and improved behavioral deficits in mice [40]. Several classes of drugs such as corticosteroids, polyphenols, alkaloids, antioxidants, and other biological compounds have been reported to exert neuroprotective effects in AD models by interfering with NF-κB signaling [10,12]. For example, a study showed that curcuminoid treatment of peripheral blood mononuclear cells collected from AD patients led to a decreased expression of NF-κB and BACE1, and consequently, Aβ clearance [41]. Additionally, a polyphenolic compound, resveratrol, markedly reduced NF-κB signaling stimulated by Aβ in glial cells and reduced neuronal death [42]. In APP/PS-1 mice, forsythoside B (FTS•B) exerts an anti-NF-κB effect and reduces Aβ plaque formation, tau phosphorylation, and microglial activation, leading to improvement in cognitive function [43]. Similarly, NF-κB signaling components were identified as top upstream regulators in tau-stimulated microglia. Consequently, the NF-κB inhibitor, TPCA-1, significantly reduced phosphorylated tau released from microglia and resulted in the rescue of tau-associated learning and memory deficits [44]. Additionally, Lindsay and colleagues evaluated the suppressive potential of a NF-κB peptide drug—glucocorticoid induced leucine zipper (GILZ) analog, or GA—on an AD mouse model. GA is known to prevent NF-κB nuclear translocation by binding to its transactivation domain. In this study, it was shown that GA reduces Aβ plaques, inhibits NF-κB activation and inflammatory cytokines in hippocampus, and suppresses gliosis in the brain of 5xFAD mice [45]. Another rising area of NF-κB involvement in AD is epigenetics. For example, both protein arginine methyltransferase 5 (PRMT5) and lysine demethylase F-box leucin rich protein 11 (FBXL11, also named KDM2A) have been reported by our lab as the novel regulators of NF-κB in cancer cells [46,47,48,49,50]. Though there are sporadic reports on the topic of PRMT5 so far, the role of PRMT5 in AD is still elusive [51]. Ultimately, the study of NF-κB regulation by epigenetic enzymes in AD and the investigation of small molecules that perturb their activities may become a prolific area of research [47].

Currently, the exploration of NF-κB inhibitors for AD treatment is yet to generate widespread interest. As a result, there are very few NF-κB-based therapies for AD in clinical trials. For example, Etanercept, a TNF inhibitor known to inhibit NF-κB signaling, which reached phase II clinical trials, failed to improve cognitive and behavioral measures that are associated with AD [52]. This could be due to the low sample size, considering that 41 patients were enrolled in the study. Thus, the underlying molecular heterogeneity in AD progression may have not been captured, including the cell-specific function of NF-κB and its mechanism of action at different stages of AD pathology. Another clinical trial [NCT03918616] evaluating the efficacy of P2X7 purinergic receptor blockers and memantine and dopamine receptor-agonists included a change in NF-κB activity as a primary outcome measure. The P2X7 receptor is overexpressed on microglial cells and oligodendrocytes in AD, and it works in collaboration with NLRP3 inflammasome and NF-κB signaling pathway to stimulate proinflammatory cytokine release [6]. This demonstrates the pivotal role that NF-κB plays in neuroinflammation in AD. Collectively, the above examples are compelling preclinical evidence that suggests the need for the comprehensive understanding of NF-κB activation and its interplay with other factors that contributes to AD progression. 

Besides the NF-κB signaling pathway, several hypotheses and targets have been identified to ameliorate AD progression. The widely acclaimed amyloid cascade hypothesis (ACH) postulates that Aβ acts as the main upstream mediator in AD pathogenesis [53]. One strategy used under the ACH involves promoting Aβ clearance. As a result, one of the earliest trials in AD treatment involved the development and testing of an Aβ vaccine. With a sample size of 30 patients, the study by Hock et al. observed that 20 out of the 24 patients who received treatment had an immune response against Aβ and exhibited lesser impaired cognition compared to the placebo group over a year-long span [54]. However, in a separate study, this approach came with setbacks when tested in a larger cohort, as 17 out of 300 patients developed meningoencephalitis [55]. Additionally, monoclonal antibodies such as aducanumab, gantenerumab, and cerenezumab, which target Aβ, reached phase III clinical trials. Nevertheless, several of those trials were halted due to the inefficacy of the drugs in slowing neurodegeneration [56,57,58]. Notably, the drug aducanumab (trade name: Aduhelm^TM^) was initially approved by the FDA based on the drug’s ability to breakdown Aβ aggregates, a common surrogate endpoint for AD. However, the FDA approval for aducanumab was later reversed due to multiple factors, including inefficacy to improve cognitive function, high costs, and occurrence of several adverse events [59]. The list of other failed drugs in clinical trials has been comprehensively summarized in another review [60]. Similarly, reducing Aβ production offers a promising strategy to stall AD progression. However, several successful preclinical β- and γ-secretase inhibitors, such as verubecestat and semagacestat, have encountered unprecedented failures in different stages of clinical trials. These drugs fail to improve cognitive decline despite their remarkable pharmacokinetic profile [36,61]. The futility of the above classes of drugs in treating AD based on ACH has precipitated the exploration of other targets for drug development. New therapeutics being actively researched and undergoing development include modulating APOE4, tau, calcium signaling, mitochondrial proteins, glutamate and acetylcholine neurotransmission, and NF-κB transcription factors [10,12,23,56,62,63]. 

An overview of the drug development landscape for AD is summarized in Table 1 [41,42,43,44,45,46,47,48,49,50,51,52,53,54,55,56,57,58,59,60,61,62,63,64,65,66,67,68,69,70,71,72,73,74,75,76,77,78,79,80,81,82,83,84], indicating the effort at multiple fronts for the development of AD treatments. 

## 5. Conclusions and Perspective

In conclusion, in this review, we provide an outlook on the role of NF-κB in the pathogenesis of AD, mainly through a variety of molecular factors involved in the activation of the NF-κB signaling pathway. For instance, these include the involvement of NF-κB with β-secretase, ApoE, glutamate, miRNA, and tau protein in neurons, reactive microglia and astrocytes (Figure 2). As aforementioned, the multifactorial nature of AD development and the staggering number of failures encountered by drugs designed to dampen AD progression is indicative of the complexity of AD etiology. This has stimulated the pursuit of novel targets for drug development, including the targeting of the NF-κB signaling pathway. Out of the 33 examples listed in Table 1, there are 11 candidates known to interfere with the NF-κB signaling pathway. Among these, Etanercept (Enbrel™) [52] is currently at the most advanced clinical trial stage (phase II). This drug works mainly through the inhibition of TNF-α activity, and consequently, NF-κB signaling. Currently, it is unknown whether the other drugs outlined in Table 1 inhibit NF-κB signaling. However, considering the extensive interaction of NF-κB with the other factors that perpetuate AD phenotypes, there is a possibility that other listed drugs may exert an anti-NF-κB effect. This possibility remains to be investigated. Hence, further research into the contribution of other novel molecules to NF-κB signaling and the detection additional early biomarkers for AD risk will further broaden the chances of preventing and curing AD in patients.

## Figures and Tables

**Table 1:** An overview of the drug development landscape for AD.

Target Protein/Pathway	Drug	Mechanism of Action	Inhibition of NF-κB Signaling	Stage of Development	References/Clinical trial ID
**NF-κB**	Etanercept (Enbrel™)	Inhibits TNF-α activity, and consequently NF-κB signaling	Yes	Phase 2 clinical trial	[52] ClinicalTrials.gov Identifier: NCT01068353
	NSAIDs	Inhibits NF-κB signaling and other inflammatory pathways.	Yes	Preclinical	[13]
	SN50	Blocks NF-κB nuclear translocation	Yes	Preclinical	[64]
	AS62868	Inhibits IKKβ	Yes	Preclinical	[13,65]
	Curcumin and curcuminoids	Decreases NF-κB and BACE1 expression	Yes	Preclinical	[41]
	Resveratrol	Deacetylation of lysine 310 on p65	Yes	Preclinical	[42]
	Forsythoside B	Decreases phosphorylation of IKKα/β, IκBα, and p65 at serine 536	Yes	Preclinical	[43]
	TPCA-1	Inhibits IKKβ	Yes	Preclinical	[44]
	Glucocorticoid induced leucine zipper (GILZ) analogs	Bind to p65 transactivation domain	Yes	Preclinical	[45]
**NMDAR**	Memantine	Antagonizes NMDA receptor	Yes	FDA Approved	[66]
	AXS-05	Antagonizes NMDAR, nicotinic receptor, serotonin and norepinephrine transporters, and agonizes sigma-1 receptor.	Unknown	Phase 2/3 clinical trial	[67] ClinicalTrials.gov Identifier: NCT03226522
**Cholinergic system**	Donepezil	Inhibits acetylcholinesterase	Unknown	FDA approved	[68,69]
	Rivastigmine	Inhibits acetylcholinesterase	Unknown	FDA approved	[70]
	Galantamine	Allosterically potentiates nicotinic receptor activity and inhibits acetylcholinesterase	Unknown	FDA approved	[71]
**Amyloid-β**	ALZT-OP1 (cromolyn+ ibuprofen)	Prevents Aβ aggregation and neuroinflammation	Unknown	Phase 3 clinical trial	[72] ClinicalTrials.gov Identifier: NCT02547818
	CAD 106	Binds to Aβ to elicit immune response	Unknown	Phase 2/3 clinical trial	[72] ClinicalTrials.gov Identifier: NCT02565511
	CNP520	Inhibits BACE1	Unknown	Phase 2/3 clinical trial	[73] ClinicalTrials.gov Identifier: NCT02565511
	E2609 (Elenbecestat)	BACE inhibitor	Unknown	Phase 3 clinical trial	[74] ClinicalTrials.gov Identifier: NCT02956486
	Solanezumab, gantenerumab	Aβ monoclonal antibodies	Unknown	Phase 2/3 clinical trial	ClinicalTrials.gov Identifier: NCT01760005
**APOE**	Bexarotene	Binds to Retinoid X receptor (RXR) agonist to increase expression of APOE which facilitates Aβ clearance.	Unknown	Preclinical	[23,75]
	PH002	Corrects the structure of the APOE4 protein associated with neuropathology in AD	Unknown	Preclinical	[76]
	Aβ12–28P	Binds to APOE4 to prevent Aβ binding, inhibiting Aβ fibril formation.	Unknown	Preclinical	[76]
**Tau**	LMTX(TRx0237)	Inhibits aggregation of hyperphosphorylated tau	Unknown	Phase 3 clinical trial	ClinicalTrials.gov Identifier: NCT03446001
	BIIB080(IONIS MAPTRx)	Inhibits the translation of tau mRNA	Unknown	Phase 2 clinical trial	[56] ClinicalTrials.gov Identifier: NCT03186989
	LY3303560 (Zagotenemab)	Monoclonal antibody to tau aggregates	Unknown	Phase 2	[77] ClinicalTrials.gov Identifier: NCT03518073
	Berberine	Inhibits tau phosphorylation and NF-κB signaling	Yes	Preclinical	[78]
	NP12	Inhibits GSK-3β to reduce tau phosphorylation	Unknown	Preclinical	[79]
**Calcium signaling**	Nimodepine	Inhibits L-type Voltage-gated calcium channel (VGCC)	Unknown	Preclinical as single agent. Phase 1 clinical trial in combination with donepezil	[69,80]
	Verapamil	Blocks L-, N-, R- and T-type VGCC in 3xTg AD mice	Unknown	Preclinical	[81]
	ST101	Inhibits T-type VGCC in 3xTg AD mice	Unknown	Preclinical	[82]
**Mitochondrial proteins**	DS44170716	Inhibits mitochondrial permeability transition which mediates cell death	Unknown	Preclinical	[83]
	Mito Q, SS31, resveratrol	Targets multiple mitochondrial protein to decrease Aβ induced toxicity and oxidative stress.	Unknown	Preclinical	[84]

## Data Availability

Not applicable.

## Abbreviations

Aβ: Amyloid β peptide; AICD: Amyloid precursor protein intracellular domain; APP: Amyloid precursor protein; sAPP: soluble peptide amyloid precursor protein; CTF: C-terminal fragments of amyloid precursor protein. APOE4: apolipoprotein E type ε4, mGluR5: metabotropic glutamate receptor 5, LPS: Lipopolysaccharide, BACE1: β-site amyloid precursor protein cleaving enzyme-1, PP2A: protein phosphatase 2A, ROS: reactive oxygen species, TLR: Toll-like receptor, AGE: Advanced Glycation End product, miRNA: microRNA.
